# The conjunctival microbiome before and after azithromycin mass drug administration for trachoma control in a cohort of Tanzanian children

**DOI:** 10.3389/fpubh.2022.1015714

**Published:** 2022-10-17

**Authors:** Harry Pickering, Athumani M. Ramadhani, Patrick Massae, Elias Mafuru, Aiweda Malisa, Kelvin Mbuya, William Makupa, Tara Mtuy, Tamsyn Derrick, Joanna Houghton, Robin L. Bailey, David C. W. Mabey, Matthew J. Burton, Martin J. Holland

**Affiliations:** ^1^Department of Clinical Research, London School of Hygiene and Tropical Medicine, London, United Kingdom; ^2^Kilimanjaro Christian Medical Centre, Moshi, Tanzania; ^3^National Institute for Health Research (NIHR) Biomedical Research Centre, Moorfields Eye Hospital NHS Foundation Trust and UCL Institute of Ophthalmology, London, United Kingdom

**Keywords:** trachoma, *Chlamydia trachomatis*, azithromycin, mass drug administration, microbiome, ocular, bacteria

## Abstract

**Background:**

Trachoma, caused by ocular infection with *Chlamydia trachomatis*, is a neglected tropical disease that can lead to blinding pathology. Current trachoma control programmes have successfully used mass drug administration (MDA) with azithromycin to clear *C. trachomatis* infection and reduce transmission, alongside promoting facial cleanliness for better personal hygiene and environmental improvement. In areas of low-trachoma endemicity, the relationship between *C. trachomatis* infection and trachomatous disease weakens, and non-chlamydial bacteria have been associated with disease signs.

**Methods:**

We enrolled a cohort of children aged 6–10 years from three adjacent trachoma endemic villages in Kilimanjaro and Arusha regions, Northern Tanzania. Children were divided into four clinical groups based on the presence or absence of ocular *C. trachomatis* infection and clinical signs of trachomatous papillary inflammation (TP). To determine the impact of treatment on the ocular microbiome in these clinical groups, we performed V4-16S rRNA sequencing of conjunctival DNA from children 3–9 months pre-MDA (*n* = 269) and 3 months post-MDA (*n* = 79).

**Results:**

*Chlamydia trachomatis* PCR-negative, no TP children had the highest pre-MDA ocular microbiome alpha diversity, which was reduced in *C. trachomatis* infected children and further decreased in those with TP. Pre-MDA, *Haemophilus* and *Staphylococcus* were associated with *C. trachomatis* infection with and without concurrent TP, while *Helicobacter* was increased in those with TP in the absence of current *C. trachomatis* infection. Post-MDA, none of the studied children had ocular *C. trachomatis* infection or TP. MDA increased ocular microbiome diversity in all clinical groups, the change was of greater magnitude in children with pre-MDA TP. MDA effectively reduced the prevalence of disease causing pathogenic non-chlamydial bacteria, and promoted restoration of a normal, healthy conjunctival microbiome.

**Conclusion:**

We identified *Helicobacter* as a non-chlamydial bacterium associated with the clinical signs of TP. Further investigation to determine its relevance in other low-endemicity communities is required. MDA was shown to be effective at clearing *C. trachomatis* infection and other non-chlamydial ocular pathogens, without any detrimental longitudinal effects on the ocular microbiome. These findings suggest that azithromycin MDA may be valuable in trachoma control even in populations where the relationship between clinical signs of trachoma and the prevalence of current ocular *C. trachomatis* infection has become dissociated.

## Introduction

Trachoma is a neglected tropical disease caused by ocular infection with *Chlamydia trachomatis*. The WHO-endorsed SAFE (surgery, antibiotics, facial cleanliness, and environmental improvement) strategy (1) for trachoma control has been implemented across the globe for 20 years, significantly reducing trachoma endemicity in many countries by stopping, or limiting, transmission of *C. trachomatis*.

Numerous studies have shown a strong association between detection of *C. trachomatis* and active trachoma disease signs, however the relationship between them is consistently weaker in settings of lower trachoma endemicity (2–5). While in some scenarios this is likely in part due to lower bacterial loads, there is considerable evidence of non-chlamydial bacteria being associated with trachomatous disease (2, 3, 6), particularly in the presence of the longer-term conjunctival scarring sequelae that lead to blindness (7, 8). In support of this, multiple studies have identified trachomatous papillary inflammation (TP) as a driver of incident and progressive conjunctival scarring, independent of *C. trachomatis* infection (9, 10).

Azithromycin MDA is effective at reducing prevalence of ocular *C. trachomatis* infection (1, 11) without giving rise to antibiotic-resistant *C. trachomatis* (12–15). However, it can drive antibiotic resistance in other bacteria, both in the nasopharynx and the gut (16–20). As well as promoting acquisition of antibiotic resistance, azithromycin MDA has consistently been shown to lead to short-term changes in the overall composition of the gut microbiota (18, 21), with variable evidence of longer-term perturbations (22, 23). As yet, no studies have explored the impact of azithromycin MDA for trachoma on the ocular microbiome.

In this study, to identify non-chlamydial bacteria associated with TP and how they're impacted by azithromycin MDA, we profiled the ocular microbiome of children from three trachoma-endemic villages in Kilimanjaro and Arusha regions of northern Tanzania 3 to 9 months pre-MDA and again 3 months post-MDA.

## Materials and methods

### Ethical approval

This study was reviewed and approved by the Tanzanian National Institute for Medical Research Ethics Committee, the Kilimanjaro Christian Medical Centre Ethics Committee, and the London School of Hygiene & Tropical Medicine Ethics Committee. It adhered to the tenets of the Declaration of Helsinki.

### Study population and clinical signs of trachoma

The details of the study design and population have been described previously (24). Briefly, the study was conducted in three adjacent trachoma endemic villages in Kilimanjaro and Arusha regions, Northern Tanzania. In January 2012, we recruited a cohort of children aged 6–10 years from these villages. The cohort was subsequently followed-up every 3 months for 4 years to investigate the pathogenesis of conjunctival scarring. At each visit, the left eye of each child was graded for trachoma by an ophthalmic nurse using the 1981 WHO Detailed Trachoma Grading System (FPC) (25), and a photograph was taken of the conjunctiva. For the purpose of this study, we consider that papillary hypertrophy scores of both two (P2) and three (P3) represent clinically significant papillary inflammation and refer to this as “TP.” Progressive scarring was subsequently determined from photographs by an ophthalmologist experienced in using a detailed scarring grading system (7), as described previously (10). Two conjunctival swabs were collected and placed into a dry tube and one containing RNAlater (Life Technologies). Air control swabs were collected after every 50 samples by passing a swab 10 cm from a participant's everted eye, these were labeled and processed identically to participant samples. All residents of the three villages received three rounds of annual mass antibiotic treatment with oral azithromycin (and topical tetracycline ointment for infants under 6 months and pregnant women), during the course of the longitudinal study.

### DNA extraction and quantification

DNA was extracted from the conjunctival swab stored in RNALater using a DNA/RNA Purification Kit (Norgen Biotek Corp), following the manufacturer's instructions. Extracted DNA was quantified on a Qubit 2.0 Fluorometer (Invitrogen) with high-sensitivity reagents (Life Technologies), following the manufacturer's instructions.

### 16S rRNA PCR and sequencing

The details of 16S rRNA amplification and sequencing have been described previously (26). Briefly, DNA libraries were prepared by amplifying a ~390 bp V4 region of the 16S rRNA gene in genomic DNA. Each PCR run included a mock bacterial community (composed of DNA from *Haemophilus influenza, Moraxella catarrhalis* and *Staphylococcus epidermis*), which acted as a positive control, and a no-template control (molecular-grade water). Purified amplicons were subjected to a second, barcoding PCR to enable downstream multiplexing. The barcoded amplicons were again purified using AMPure XP beads (Beckman Coulter) and then quantified on a Qubit 2.0 Fluorometer (Invitrogen) with high-sensitivity reagents (Life Technologies) and thereafter pooled in equimolar amounts. A PhiX-spiked DNA library was then assayed by 2 × 300 bp paired-end sequencing on the Illumina MiSeq platform for a total of 600 cycles. Anonymized V4-16S sequencing data are available from the Sequence Read Archive (SRA) at the National Center for Biotechnology Information (NCBI) under accession number PRJNA815053.

### Data quality control and analysis

Raw reads generated by MiSeq were error-corrected and filtered using DADA2 (27) through QIIME2 (28). Filtered reads were clustered *de novo* into Operational Taxonomic Units (OTUs) at 97% sequence similarity. OTUs were then assigned taxonomy using a Naive Bayes classifier trained on the SILVA 16S database. Both processes were performed with QIIME2. Manual filtering of classified OTUs was performed using R as described below. OTUs were retained if they had been classified as bacteria, had a genus-level classification and constituted >0.005% of the total number of reads. *Ralstonia* and *Nocardioides* were removed because they had an average abundance of >1,000 reads in the no-template (water) and air controls, respectively. Samples with < 1,000 reads were excluded. Final read counts were rarefied to 10,000 reads per sample using the R package *vegan*. Alpha diversity (Shannon's H) and beta diversity (weighted UniFrac distance) were calculated using the R package *vegan*.

### *Chlamydia trachomatis* PCR

To detect *C. trachomatis*, we extracted DNA from conjunctival swab samples stored in dry tubes using the PowerSoil DNA Isolation Kit (Mo Bio Laboratories), according to the manufacturer's instructions. The cells attached to the swabs were initially disrupted by bead beating to release their contents. *C. trachomatis* DNA was detected using a previously described droplet digital PCR assay (29). All samples were tested for the *C. trachomatis* plasmid and the human gene *RPP30*.

### Statistical analysis

For all downstream comparisons, samples were defined as “Ct–/TP–” (*C. trachomatis* PCR-negative, no TP), “Ct+/TP–” (*C. trachomatis* PCR-positive, no TP), “Ct–/TP+” (*C. trachomatis* PCR-negative, signs of TP) or “Ct+/TP+” (*C. trachomatis* PCR-positive, signs of TP). Alpha diversities were compared by linear regression, adjusting for age. Beta diversities were compared by PERMANOVA with 1,000 permutations using the R package *vegan*. Prevalence (read count >0) and rarefied abundances were compared between clinical groups by binomial logistic regression. *P*-values were corrected for multiplicity of testing using the Benjamini-Hochberg procedure.

## Results

### Pre-MDA

From the complete cohort of 666 children, 633 had DNA available for sequencing prior to MDA. Based on clinical trachoma grading and *C. trachomatis* PCR testing, children were classified as “Ct–/TP–,” “Ct+/TP–,” “Ct–/TP+” or “Ct+/TP+.” We selected all available children with Ct+TP– (*n* = 48), the smallest clinical group, then selected age, sex, and village-matched children from the remaining three clinical groups. After removing samples with low quantity or quality of DNA, and post-sequencing quality control, we studied ocular bacterial microbiome profiles of 269 children prior to azithromycin MDA by V4-16S rRNA sequencing (Table 1). *C. trachomatis* plasmid load, unquantifiable in Ct-/TP- and Ct-/TP+ groups, was significantly higher in Ct+/TP+ compared with Ct+/TP–. Conjunctival scarring grades were higher in Ct–/TP+ and Ct+/TP+.

**Table 1:** Clinical information and demographics of the four study groups pre-MDA.

**Variable**	**Ct–/TP–** **(*n* = 159)**	**Ct+/TP–** **(*n* = 44)**	**Ct–/TP+** **(*n* = 29)**	**Ct+/TP+** **(*n* = 37)**	***p*-value**
Median age in years (range)	8 (6-13)	7 (6-12)	7 (6-11)	7 (6-11)	0.070
Female (%)	88 (55.3%)	20 (45.5%)	16 (55.2%)	23 (62.2%)	0.497
Median Ct plasmid load (range)	–	2.28 (0.06–1717.48)	–	30.19 (0.07–47343.83)	0.017
**Village**					0.103
1	49 (30.8%)	12 (27.3%)	13 (44.8%)	15 (40.5%)	
2	21 (13.2%)	7 (15.9%)	5 (17.2%)	10 (27.0%)	
3	89 (56.0%)	25 (56.8%)	11 (37.9%)	12 (32.4%)	
**Scarring grade**					0.654 × 10^−4^
0	123 (77.4%)	31 (75.0%)	14 (48.3%)	17 (45.9%)	
1	31 (19.5%)	11 (25.05%)	11 (37.9%)	16 (43.2%)	
2	5 (3.1%)	0 (0.0%)	4 (13.8%)	4 (10.8%)	

**Figure 1:**
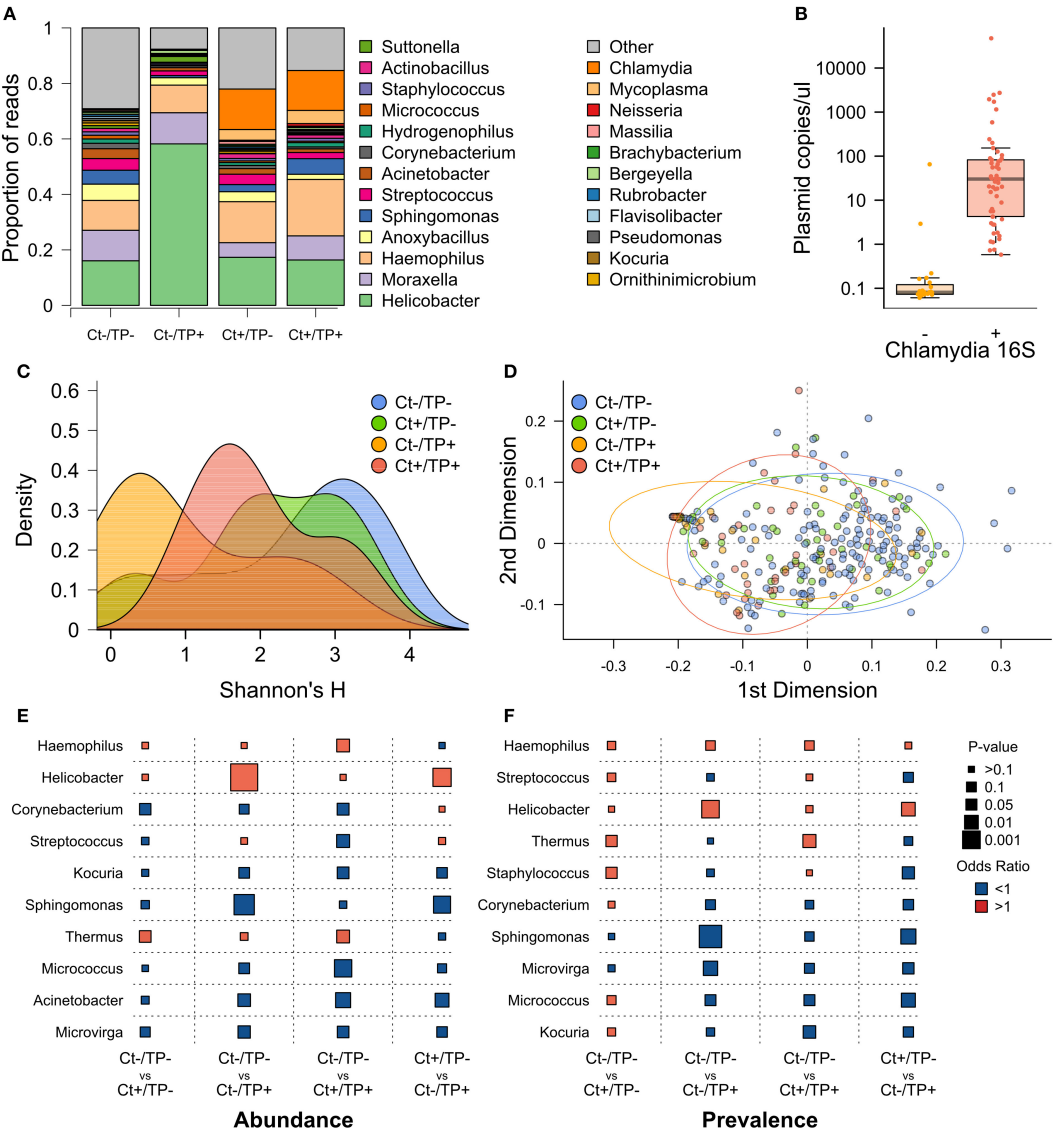
Pre-MDA ocular microbiome by Ct and TP status. **(A)** Genera with ≥1% relative abundance, all genera below < 1% are grouped as “Other.” **(B)** Ct plasmid load, quantified by PCR, in children who had detectable Chlamydia reads by 16S rRNA PCR (+) or not (–). **(C)** Alpha diversity, measured by Shannon's H, by group. **(D)** PCA of beta diversity, measured by weighted uniFrac distance. **(E)** Logistic regression comparing genera abundance between indicated clinical groups, odds ratio (color of points; red >1, blue < 1) and *p*-value (size of points). **(F)** Logistic regression comparing genera prevalence (read count >0) between indicated clinical groups.

Visual inspection of genera with ≥1% group-level abundance (Figure 1A) showed Ct+/TP– and Ct+/TP+ groups were similar but were separated from the Ct–/TP– groups by the presence of *Chlamydia* and increased abundance of *Mycoplasma*. The CT–/TP+ group had increased abundance of *Helicobacter* and a reduced abundance of “Other” genera (“Other” constituting a group consisting of each genera < 1% overall abundance). All children in which the genera *Chlamydia* was detected by V4-16S rRNA amplicon sequencing were *C. trachomatis* PCR-positive (*n* = 57), however, a further 24 Ct PCR-positive children had zero-read counts for Chlamydia by 16S rRNA-amplicon sequencing (Figure 1B). *C. trachomatis* plasmid load was significantly higher in 16S rRNA-positive individuals (*p* = 0.009).

Alpha diversity, quantified by Shannon's H, was highest in Ct–/TP– children (Figure 1C), this was also true for Simpson's D. This difference was significant for Ct–/TP+ [*p* = 0.009, scarring-grade adjusted p (adj.p) =0.011] but not Ct+/TP– (*p* = 0.482, adj.p = 0.552) or Ct+/TP+ (*p* = 0.325, adj.p = 0.398). Ct–/TP+ alpha diversity was non-significantly lower compared with Ct+/TP– (*p* = 0.0790, adj.p = 0.080) and Ct+/TP+ (*p* = 0.113, adj.p = 0.114). Beta diversity, quantified by weighted UniFrac distance, was clearly different in Ct-/TP+ and to a lesser extent Ct+/TP+ (Figure 1D). PERMANOVA analysis found the differences in beta diversity to be significant (*p* = 0.001, adj.p = 0.001). Ct–/TP– and Ct+/TP– were not different.

Genera relative abundance (Figure 1E) and prevalence (Figure 1F) in Ct+/TP–, Ct–/TP+ and Ct+/TP+ were compared with the Ct–/TP– group. Additionally, we compared Ct+/TP– with Ct–/TP+. Ct+/TP– had significantly increased abundance (*p* = 0.034) and prevalence (*p* = 0.042) of *Thermus*, increased prevalence of *Staphylococcus* (*p* = 0.044) and decreased abundance of *Corynebacterium* (*p* = 0.040). Ct+/TP+ also had significantly increased *Thermus* (abundance *p* = 0.017, prevalence *p* = 0.015), as well as *Haemophilus* abundance (*p* = 0.018), with decreased abundance of multiple genera, most significantly *Acinetobacter* (*p* = 0.005), Micrococcus (*p* = 0.001) and *Streptococcus* (*p* = 0.016). Ct–/TP+ only had significantly increased abundance (*p* = 0.393 × 10^−5^) and prevalence of *Helicobacter* (*p* = 0.001), but had decreased abundance and prevalence of multiple genera, most significantly *Acinetobacter* (abundance *p* = 0.019) and *Sphingomonas* (abundance *p* = 0.208 × 10^−3^, prevalence *p* = 0.608 × 10^−4^). Comparison of Ct+/TP– and Ct–/TP+ reinforced these differences, highlighted by increased *Helicobacter* in Ct-/TP+ (abundance *p* = 0.704 × 10^−3^, prevalence *p* = 0.011) and reduced *Sphingomonas* (abundance *p* = 0.001, prevalence *p* = 0.004).

### Post-MDA

Seventy-nine of 269 children profiled pre-MDA had DNA available to profile their ocular microbiome 3 months post-MDA. All 79 children were *C. trachomatis* PCR-negative and had no evidence of clinical signs of TP post-MDA, therefore we compared post-MDA changes by pre-MDA clinical group. The four clinical groups as defined by their pre-MDA status, were still equivalent in age, sex and resident village (Table 2). There was marginal evidence of higher conjunctival scarring grade in Ct+/TP+. All 79 children received azithromycin, while community-wide treatment coverage was 68.7% (30).

**Table 2:** Clinical information and demographics of the four study groups pre-MDA.

**Variable**	**Ct–/TP–** **(*n* = 58)**	**Ct+/TP–** **(*n* = 11)**	**Ct–/TP+** **(*n* = 8)**	**Ct+/TP+** **(*n* = 11)**	***p*-value**
Median age in years (range)	10 (8–15)	10 (8–15)	10 (9–13)	10 (8–14)	0.596
Female (%)	29 (50.0%)	3 (27.3%)	5 (62.5%)	6 (54.5%)	0.406
**Village**					0.675
1	9 (15.5%)	2 (18.2%)	1 (12.5%)	4 (36.4 %)	
2	4 (6.9%)	2 (18.2%)	1 (12.5%)	1 (9.1%)	
3	45 (77.6%)	7 (63.6%)	6 (75%)	6 (54.5%)	
**Scarring grade**					0.032
0	49 (84.5%)	8 (72.7%)	5 (62.5%)	4 (36.4%)	
1	7 (12.1%)	3 (27.3%)	2 (25.0%)	6 (54.5%)	
2	2 (3.5%)	0 (0.0%)	1 (12.5%)	1 (9.1%)	

**Figure 2:**
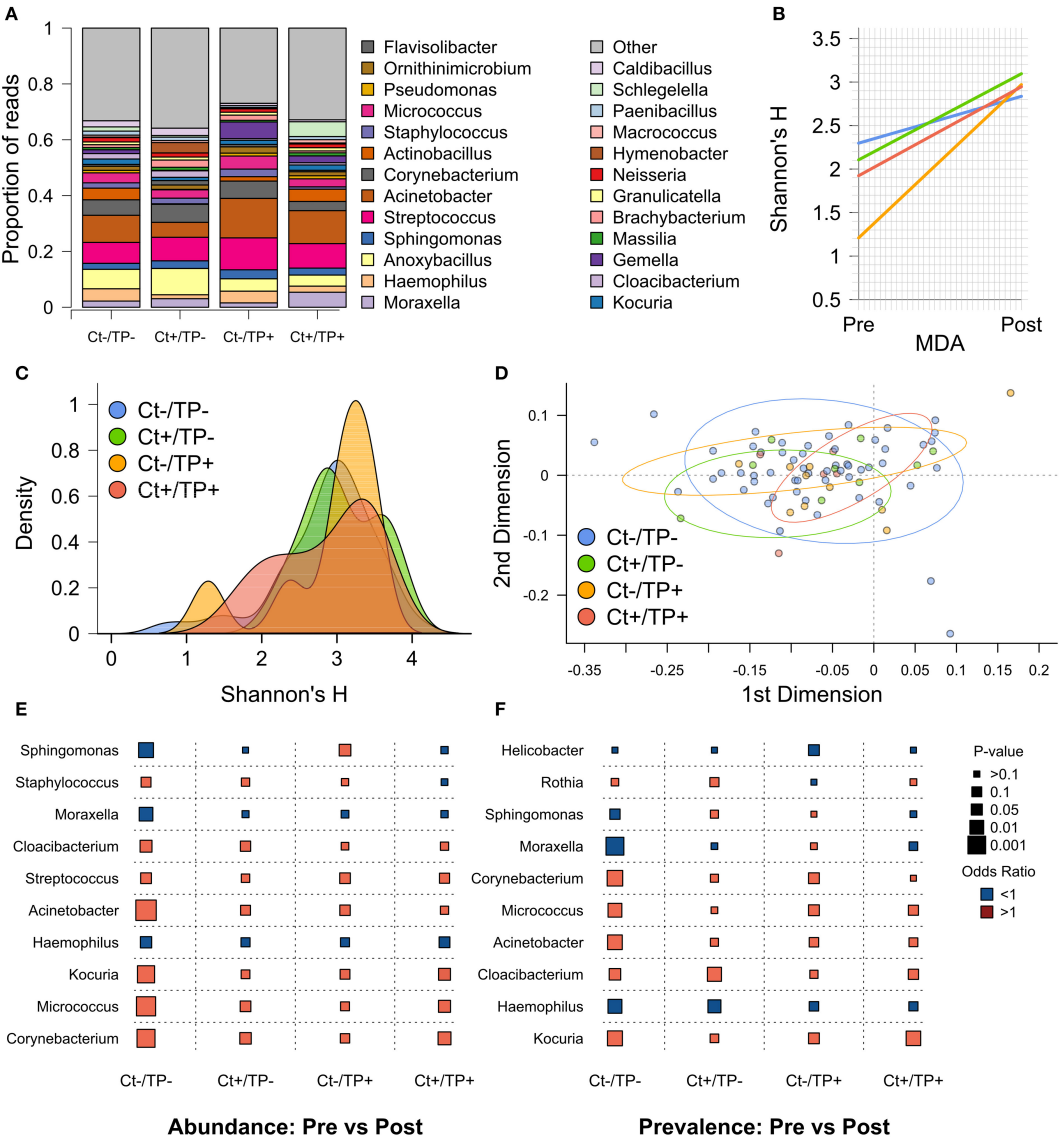
Post-MDA ocular microbiome by pre-MDA Ct and TP status. **(A)** Genera with ≥1% relative abundance, all genera below < 1% are grouped as “Other.” **(B)** Change in Shannon's H from pre- to post-MDA. **(C)** Alpha diversity, measured by Shannon's H, by group. **(D)** PCA of beta diversity, measured by weighted uniFrac distance. **(E)** Logistic regression comparing genera abundance from pre- to post-MDA for indicated clinical groups, odds ratio (color of points; red >1, blue < 1) and *p*-value (size of points). **(F)** Logistic regression comparing genera prevalence (read count >0) from pre- to post-MDA for indicated clinical groups.

Visual inspection of genera with ≥1% group-level abundance showed no clear differences between the groups at the post-MDA timepoint (Figure 2A). Alpha diversity, quantified by Shannon's H, significantly increased from pre- to post-MDA for all four groups (Figure 2B), this was also true for Simpson's D. The greatest magnitude of change was in Ct–/TP+ (linear regression coefficient [coef] = 0.602, standard error [SE] = 0.153), Ct+/TP– (coef = 0.323, SE = 0.119) and Ct+/TP+ (coef = 0.287, SE = 0.106) were similar, with the smallest increase for Ct–/TP– (coef = 0.170, SE = 0.061).

Alpha diversity post-MDA was not significantly different between any of the groups (Figure 2C). Although a subset of Ct–/TP+ children still had lower alpha diversity, the same was true to a lesser extent for Ct+/TP+. Beta diversity post-MDA, quantified by weighted UniFrac distance, was similar between groups (Figure 2D). PERMANOVA analysis found slight differences in beta diversity (*p* = 0.033), appearing to be driven primarily by Ct+/TP+.

To identify any genera driving the increase in diversity seen after MDA, we compared relative abundance (Figure 2E) and prevalence (Figure 2F) of genera between pre- and post-MDA for each of the clinical groups. The Ct-/TP- group had significant changes in many genera post-MDA, most notably increased *Acinetobacter* (abundance *p* = 0.206 × 10^−3^, prevalence *p* = 0.006), *Corynebacterium* (abundance *p* = 0.871 × 10^−3^, prevalence *p* = 0.004) and *Micrococcus* (abundance *p* = 0.435 × 10^−3^, prevalence *p* = 0.010), with decreased *Haemophilus* (abundance *p* = 0.043, prevalence *p* = 0.009), *Moraxella* (abundance *p* = 0.011, prevalence *p* = 0.931 × 10^−3^) and *Sphingomonas* (abundance *p* = 0.005). Both Ct+/TP– and Ct+/TP+ had increased abundance of *Corynebacterium* (Ct+/TP– *p* = 0.038, prevalence *p* = 0.017), with additionally increased prevalence of *Cloacibacterium* (*p* = 0.009) in Ct+/TP–, and *Micrococcus* (abundance *p* = 0.028) and *Kocuria* (abundance *p* = 0.030, prevalence *p* = 0.007) in Ct+/TP+. Ct+/TP– also had decreased prevalence of *Haemophilus* post-MDA (*p* = 0.016). *Sphingomonas* abundance was increased in Ct–/TP+ (*p* = 0.035). Since increased *Corynebacterium* abundance has previously been found in scarring trachoma, we tested for association between 4 year scarring progression and *Corynebacterium* abundance. Neither change in abundance of *Corynebacterium* from pre- to post-MDA (*p* = 0.652) nor abundance post-MDA (*p* = 0.392) were associated with progressive scarring trachoma (Table 3).

**Table 3:** No evidence of an association between Coryebacterium and scarring progression.

***Corynebacterium***	Non-progressors (*n* = 61)*	**Scarring progressors (*n* = 22)**	***p*-value**
Median change in percent abundance from pre- to post-MDA (IQR)	2.3 (0.0–6.1)	2.7 (1.2–5.1)	0.652
Median percent abundance post-MDA (IQR)	3.9 (1.9–8.3)	2.7 (1.5–6.0)	0.392

*Five children had photographs not suitable for grading scarring progression and were not included.

## Discussion

This study profiled the ocular microbiome of children from three trachoma-endemic villages in Kilimanjaro and Arusha regions of northern Tanzania pre- and post-MDA with azithromycin. Ocular *C. trachomatis* infection and TP, both alone and in combination (Ct+/TP+), were associated with reduced alpha diversity and changes in individual genera. Notably *Acinetobacter* and *Sphingomonas* were reduced compared with Ct–/TP- children, with increased *Thermus* in both Ct+/TP– and Ct+/TP+, and increased *Helicobacter* in Ct–/TP+. Post-MDA there was no *C. trachomatis* infection or clinical signs of TP, alpha diversity was increased in all groups and ocular microbial profiles were equivalent. MDA was associated with consistent increases in *Corynebacterium, Kocuria*, and *Micrococcus* regardless of pre-MDA clinical group, as well as decreased abundance of *Helicobacter* and *Haemophilus*. Notably, MDA was associated with decreased prevalence of *Sphingomonas* in Ct–/TP– children post-MDA but increased in those with Ct–/TP+.

This study found that TP in the absence of *C. trachomatis* infection, had a greater effect on the ocular microbiome than infection alone or in combination with TP. This may be in part due to timing of sampling since TP is known to persist after clearance of infection (4). *C. trachomatis*-induced changes in the ocular microbiome may continue to develop, or at least be maintained, after clearance. In support of this, Ct+/TP–, Ct+/TP+, and Ct–/TP+ had similar profiles for several genera. Specifically, *Acinetobacter* was decreased in both Ct+/TP+ and Ct–/TP+, while *Thermus* was increased in both Ct+/TP– and Ct+/TP+. Alternatively, there may be non-chlamydial causes of ocular inflammation, multiple studies have reported such associations, most frequently for *Streptococcus, Haemophilus*, and *Corynebacterium* (2, 3, 6–8). We found, Ct+/TP- and Ct+/TP+ groups had increased *Staphylococcus* and *Haemophilus*, respectively, while Ct-/TP+ had increased *Helicobacter*. There is a growing literature of *Helicobacter pylori* infection associated with chronic eye diseases (31), particularly diseases in which oxidative stress is an important factor, but the direction of causation remains unknown. This is also true in our study, *Helicobacter* infection may be driven or facilitated by changes to the conjunctiva following *C. trachomatis* infection and/or TP, or longitudinal changes due to scarring trachoma, alternatively, it may itself be causing inflammation. Given that TP is consistently found to be associated with both new and increasing conjunctival scarring (9, 10), it is plausible that continued presence of non-*C. trachomatis* pathogens such as *Helicobacter* in the conjunctiva, in the absence of treatment, may drive persistent immune responses leading to scarring pathology.

Importantly, azithromycin MDA effectively cleared *Helicobacter* from the studied population, despite high prevalence pre-MDA. Similarly, Ct+/TP+-associated *Haemophilus* was decreased post-MDA, as well as *Moraxella* which is known to cause conjunctival inflammation (32, 33). None of the studied children had ocular *C. trachomatis* infection or clinical signs of TP 3 months post-MDA in addition to similar microbiome profiles, in terms of alpha and beta diversity, across all children following treatment regardless of pre-MDA clinical status. MDA also increased microbial diversity in all children, with the greatest magnitude observed in Ct-/TP+. Azithromycin MDA has been proven to be effective at clearing ocular *C. trachomatis* infection (11) and is clearly shown in this data and the larger study cohort, where *C. trachomatis* infection prevalence decreased from 11.6% pre-MDA to 1.3% post-MDA (30). However, this is the first study to determine the effects of MDA on non-chlamydial bacteria and the broader conjunctival microbiome. These results show that MDA decreases the abundance and/or prevalence of potentially pathogenic bacteria and restores a healthy microbiome in children with *C. trachomatis* and/or TP, without causing detrimental changes in Ct-/TP- children. MDA was associated with consistent increases in *Corynebacterium* and *Micrococcus*, both of which are primarily commensal residents of the skin and conjunctiva (34, 35). Notably, multiple studies have found an association between *Corynebacterium* and scarring trachoma (6, 36), with links to gene expression patterns involved in responses to the microbiota, including mucins (6). These studies were not designed to demonstrate whether increased *Corynebacterium* abundance was a result or cause of scarring trachoma. The finding that MDA consistently increases the abundance of *Corynebacterium*, regardless of pre-treatment clinical status, would suggest it is unlikely to be driving pathological changes to the conjunctiva in this population, supported by the lack of association between abundance of *Corynebacterium* post-MDA and progressive scarring trachoma.

In conclusion, *C. trachomatis* and TP alone or in combination, were both associated with pre-MDA perturbations in the ocular microbiome. *Helicobacter* was identified as a novel non-chlamydial correlate of TP, alongside previously defined pathogens such as *Haemophilus* and *Staphylococcus*. Azithromycin MDA cleared ocular *C. trachomatis* infection from the studied individuals, as well as TP. Furthermore, MDA cleared or reduced the prevalence of numerous potential pathogens, including those highlighted above, while restoring individual genera indicative of a normal, healthy ocular microbiota.

## Data availability statement

The datasets presented in this study can be found in online repositories. The names of the repository/repositories and accession number(s) can be found at: https://www.ebi.ac.uk/ena/browser/view/PRJEB46956?show=reads, PRJEB46956.

## Ethics statement

The studies involving human participants were reviewed and approved by Tanzanian National Institute for Medical Research Ethics Committee Kilimanjaro Christian Medical Centre Ethics Committee London School of Hygiene & Tropical Medicine Ethics Committee. Written informed consent to participate in this study was provided by the participants' legal guardian/next of kin.

## Author contributions

AR, RB, DM, MB, and MH contributed to study design. HP, AR, PM, EM, AM, KM, WM, TM, TD, JH, MB, and MH contributed to data collection. HP, MB, and MH contributed to data analysis. All authors interpreted the findings, contributed to writing the manuscript, and approved the final version for publication.

## Funding

This work was supported by the Wellcome Trust, grant numbers: 098481/Z/12/Z to MB and 093368/Z/10/Z to MB and MH.

## Conflict of interest

The authors declare that the research was conducted in the absence of any commercial or financial relationships that could be construed as a potential conflict of interest.

## Publisher's note

All claims expressed in this article are solely those of the authors and do not necessarily represent those of their affiliated organizations, or those of the publisher, the editors and the reviewers. Any product that may be evaluated in this article, or claim that may be made by its manufacturer, is not guaranteed or endorsed by the publisher.
